# Naming the genus Marivivens and its species: etymological considerations

**DOI:** 10.1099/ijsem.0.006770

**Published:** 2025-04-30

**Authors:** Aharon Oren, Marko Kostovski, Bernhard Schink, Stefano Ventura

**Affiliations:** 1 Institute of Life Sciences, The Hebrew University of Jerusalem, The Edmond J. Safra Campus, 9190401 Jerusalem, Israel; 2 Institute of Microbiology & Parasitology, Faculty of Medicine-Skopje, University Ss Cyril and Methodius in Skopje, 1000 Skopje, Republic of North Macedonia; 3 Department of Biology, University of Konstanz, D-78457 Konstanz, Germany; 4 Research Institute on Terrestrial Ecosystems, National Research Council of Italy, 50019 Sesto Fiorentino, Italy; 5 National Biodiversity Future Center, Palermo, Italy

**Keywords:** adjectives, gender, *Marivivens*, nomenclature, participles

## Abstract

The name *Marivivens marinum*, published as sp. nov. by Kang *et al.* (*Int J Syst Evol Microbiol* 2025;75 : 006735) contravenes Rule 12 c(1) of the International Code of Nomenclature of Prokaryotes (ICNP). Albeit the authors of the genus name *Marivivens* Park *et al.* 2016 did not specify the gender of the name, the epithet *donghaensis* of the type species was given the masculine gender, and so was the epithet of *Marivivens geojensis* (Lee *et al.* 2019) Qu *et al*. 2022. Therefore, we here correct the name *Marivivens marinum* to *Marivivens marinus* corrig. We also give examples of the correct use of present participles as genus names, based on the rules of the ICNP and on past usage in the nomenclature of prokaryotes.

In the March issue of the *International Journal of Systematic and Evolutionary Microbiology (IJSEM)*, Kang *et al.* [1] described a new species of the genus *Marivivens* Park *et al*. 2016 [2] as *Marivivens marinum* sp. nov. Thus, the specific epithet was assigned the neuter gender.

Following Rule 12 c(1) of the International Code of Nomenclature of Prokaryotes (ICNP) [3], the gender of adjectival epithets must agree with the gender of the generic name. The authors of the generic name *Marivivens* did not assign a gender, and they presented the etymology as ‘N.L. part. adj. *Marivivens* living in the sea’ [2], contravening Rule 10a, which states that the name of a genus … is a noun or an adjective used as a noun. For that reason, the List of Prokaryotic names with Standing in Nomenclature (LPSN) presented the etymology of the name as N.L. masc./fem. n. *Marivivens*, living in the sea (https://lpsn.dsmz.de/search?word=Marivivens, accessed 27 March 2025), assigning the masculine or the feminine gender to the generic name. However, the type species of the genus, *Marivivens donghaensis,* was given as masculine by its authors (N.L. masc. adj. *donghaensis* of Donghae, the Korean name for the East Sea of South Korea …) [2], and so was *Marivivens geojensis* (Lee *et al.* 2019) Qu *et al.* 2022 (N.L. masc. adj. *geojensis*, pertaining to Geoje Island) [4]. LPSN gives both adjectival epithets as N.L. masc./fem. adj. (https://lpsn.dsmz.de/species/marivivens-donghaensis and https://lpsn.dsmz.de/species/marivivens-geojensis, accessed 27 March 2025).

From the above, it must be inferred that the generic name *Marivivens* has the masculine gender, as adjectival epithets must agree in gender with the generic name [Rule 12 c(1)]. In any case, the gender cannot be neuter, as then the epithets must be *donghaense* and *geojense. Marivivens marinum* is therefore incorrect.

Manuscripts submitted for publication in the *IJSEM* are checked by the four authors of this Letter, who serve as the journal’s nomenclature reviewers (https://www.microbiologyresearch.org/content/journal/ijsem?page=editorial-board). We had seen and commented on draft versions of the Kang *et al.* 2025 paper [1] in which the authors proposed other names for the species. However, due to a technical error, they did not receive the final version with the name *Marivivens marinum* for approval. A correction of the name will be presented in the Notification List for new names of prokaryotes that have appeared in volume 75, part 3 of the *IJSEM* [5]. As clarified by Rule 27 of the ICNP, the Notification List allows for orthographic and grammatical corrections to be made [3].

Grammatically, the generic name *Marivivens* is a present participle (from *vivens*, living, derived from the Latin verb *vivere*, to live). Participles are often used as adjectives in the formation of compound generic names and in specific epithets [6]. This use is clarified in Appendix 7 of the ICNP [3], where the abbreviation part. adj. is given for ‘participle used as adjective’ with the explanation ‘To comply with Rule 12 c(1) so that a participle can be used as a specific or subspecific epithet’.

Latin present participles end on -*ans* (first conjugation) or *-ens* (second, third and fourth conjugation) and have the same form in all three genders. As generic names ending in *vivens* (third conjugation), we have the masculine *Litorisediminivivens* Park *et al.* 2016 (‘living in coastal sediment’), the feminine *Brotonthivivens* Hitch *et al.* 2022 (‘a microbe from the faeces of humans’), *Litorivivens* Park *et al.* 2015 (‘living at the seashore’) and *Suilimivivens* Hitch *et al.* 2022 (‘a microbe frequently occurring in the faeces of pigs’), and the neuter *Aestuariivivens* Park *et al.* 2015 (‘living in a tidal flat’) and *Pontivivens* Park *et al.* 2015 (‘living in the sea’). Most generic names formed as present participles were assigned the masculine gender. Examples are *Agarivorans* Kurahashi and Yokota 2004 (‘agar-devouring’), *Mangrovihabitans* Liu *et al.* 2017 (‘mangrove-inhabiting’), *Saccharedens* Lin *et al.* 2017 (‘sugar-eating’) and *Nonlabens* Lau *et al.* 2005 (‘non-gliding’). *Lacisediminihabitans* Zhuo *et al.* 2020 (‘an inhabitant of lake sediment’), *Methanomethylovorans* Lomans *et al.* 2004 (‘methane producing methyl consuming’) and *Mycetohabitans* Estrada-de Los Santos *et al.* 2018 (‘inhabitant of fungi’) were given the feminine gender by the respective authors. A few were given the neuter gender: *Citrifermentans* Waite *et al.* 2020 (‘citrate fermenting’), *Ethanoligenens* Xing *et al.* 2006 [‘ethanol-producing (bacterium)’], *Myceligenerans* Cui *et al.* 2004 (‘hyphae-forming microbe’) and *Saccharofermentans* Chen *et al.* 2010 (‘sugar-fermenting’).

To avoid further confusion, we here present the correct names and etymologies of the genus *Marivivens* and its members:

*Marivivens* Park *et al.* 2016 [2]: (Ma.ri.vi’vens. L. neut. n. *mare*, the sea; L. part. adj. *vivens*, living; N.L. masc. n. *Marivivens*, an organism living in the sea).

‘*Marivivens aquimaris*’ Kim *et al*. 2021 [7] (not validly published): (a.qui.ma’ris. L. fem. n. *aqua*, water; L. neut. n. *mare*, the sea; N.L. gen. n. *aquimaris*, of seawater).

*Marivivens donghaensis* Park *et al.* 2016 [2]: (dong.ha.en’sis. N.L. masc. adj. *donghaensis*, of Donghae, the Korean name for the East Sea of South Korea).

*Marivivens geojensis* (Lee *et al*. 2019) Qu *et al*. 2022 [4]: (ge.o.jen’sis. N.L. masc. adj. *geojensis*, pertaining to Geoje Island).

*Marivivens marinus* corrig. Kang *et al*. 2025 [1,5]: (ma.ri’nus. L. masc. adj. *marinus*, marine).

*Marivivens niveibacter* Hu *et al.* 2018 [8]: (ni.ve.i.bac’ter. L. masc. adj. *niveus*, white; N.L. masc. n. *bacter*, rod; N.L. masc. n. *niveibacter*, a white rod).

## Abbreviations:

ICNP: International Code of Nomenclature of Prokaryotes
IJSEM: International Journal of Systematic and Evolutionary Microbiology
LPSN: List of Prokaryotic names with Standing in Nomenclature
